# Assessment of Groundwater Contamination by Terbuthylazine Using Vadose Zone Numerical Models. Case Study of Valencia Province (Spain)

**DOI:** 10.3390/ijerph17093280

**Published:** 2020-05-08

**Authors:** Javier Rodrigo-Ilarri, María-Elena Rodrigo-Clavero, Eduardo Cassiraga, Leticia Ballesteros-Almonacid

**Affiliations:** Instituto de Ingeniería del Agua y Medio Ambiente, Universitat Politècnica de València (IIAMA-UPV), 46022 Valencia, Spain; marodcla@upv.es (M.-E.R.-C.); efc@dihma.upv.es (E.C.); l.ballesteros.a@gmail.com (L.B.-A.)

**Keywords:** terbuthylazine, modeling, vadose zone, organic pollutants

## Abstract

Terbuthylazine is commonly used as an herbicide to control weeds and prevent non-desirable grow of algae, fungi and bacteria in many agricultural applications. Despite its highly negative effects on human health, environmental modeling of this kind of pesticide in the vadose zone till reaching groundwater is still not being done on a regular basis. This work shows results obtained by two mathematical models (PESTAN and PRZM-GW) to explain terbuthylazine behavior in the non-saturated zone of a vertical soil column. One of the models use a one-dimensional analytical formulation to simulate the movement of terbuthylazine through the non-saturated soil to the phreatic surface. The second and more complex model uses a whole set of parameters to solve a modified version of the mass transport equation considering the combined effect of advection, dispersion and reactive transport processes. Both models have been applied as a case-study on a particular location in South Valencia Aquifer (Spain). A whole set of simulation scenarios have been designed to perform a parameter sensitivity analysis. Despite both models leading to terbuthylazine’s concentration values, numerical simulations show that PRZM-GW is able to reproduce concentration observations leading to much more accurately results than those obtained using PESTAN.

## 1. Introduction

Pesticides are “substances or organisms used to eliminate, incapacitate, modify, inhibit growth of or repel pests. They can be natural or synthetic chemicals, mixtures of these, or living organisms that act as biological control agents” [1].

Infiltration processes cause pesticides to infiltrate in the vadose zone. These transport processes are highly dependent on the concentration of pesticide used and its physicochemical characteristics and interaction with the environment. If recharge is high enough the transport process of this kind of organic pollutants from the surface may take a long time till finally reaching the aquifer. Chemical composition of pesticides and their structure justifies their ability to migrate when they are dissolved in groundwater. The mobility of an organic chemical in soil is related to the octanol–water partitioning coefficient, K_ow_. For nonionic chemicals and some other chemicals, the partitioning can be correlated to the octane partitioning coefficient [2].

Though organic chemicals, such as pesticides, are often adsorbed in the solid phase of the non-saturated part of soil, they may be disposed again under favorable conditions. In these situations, the position of the phreatic surface is a key factor to determine the time taken by the pesticide to finally reach the saturated zone of the aquifer. If the phreatic surface is deep enough these transit times can be high so the groundwater contamination problem is deferred in time [1,3].

Terbuthylazine-TBA (C_9_H_16_ClN_5_) is one of the most common herbicides used to control grass and broad-leaved weeds in a variety of situations including forestry and for controlling slime-forming algae, fungi and bacteria [4]. The IUPAC (International Union of Pure and Applied Chemistry) name for terbuthylazine (TBA) is 6-chloro-N-(1,1-dimethylethyl)-N’-ethyl-1,3,5- triazine-2,4-diamine. TBA is an herbicide that belongs to the chloro-triazine family. In plants, it acts as a powerful inhibitor of photosynthesis. The substance is taken up through both roots and leaves and is distributed throughout the plant after being taken up through the roots. This enables it to be used in both pre- and post-emergence treatment. TBA is a selective herbicide for maize, sorghum, potatoes, peas, sugar cane, vines, fruit trees, citrus, coffee, oil palm, cocoa, olives, rubber and forestry in tree nurseries and new planting. It is particularly effective against annual dicotyledons [5]. Physicochemical properties of terbuthylazine are shown in Table 1 [6].

Degradation of TBA in natural water depends on the presence of sediments and biological activity. Although studies with Rhine River and pond water estimated a half-life value higher than one year, newer findings indicate this value to be approximately 50 days [5].

Despite this, it has not been widely used yet on pesticide analysis for practical applications. Numerical modelling provides interesting alternatives to simulate fate and transport of pesticides on soil and groundwater. Some research has been done to understand the fate of organic contaminants in the non-saturated zone [7,8,9,10], and specifically in pesticides [11,12].

The objective of this work is to demonstrate the application and analyze the results obtained by two mathematical models to assess groundwater contamination by terbuthylazine using vadose-zone transport models on a vertical soil column. Results will be compared with actual concentrations of terbuthylazine which have been measured on the underlying aquifer of an agricultural site located near the town of Picassent in Valencia Province (Spain).

## 2. Description of the Study Area. The South Valencia Plain Aquifer

Following the official description of the Spanish Mediterranean aquifers in the Valencia Region [13], the aquifers of North Valencia Plain, South Valencia Plain, Buñol-Cheste and Sierra del Ave are located geographically between the towns of Puzol, to the northeast, Loriguilla to the northwest, Cortes de Pallás to the west, Antella to the southwest and Cullera to the southeast. From a geological point of view, they are located between the southeastern end of the Iberian Mountain Range, the northeast of the Baetican Mountain range and the Mediterranean Sea, in the vicinity of the coastal plain of the Gulf of Valencia. In this wide territory, two morphologically different areas can be established. The closest to the coast, where the Albufera lake is located, is occupied by modern materials and has a very smooth topography, while in the interior the relief becomes progressively more abrupt, first with the appearance of Miocene formations and, in isolation, the Mesozoic mountain ranges of La Rodana and Perenchiza, and, later, with the Jurassic and Cretaceous materials of the Iberian and Betic Mountain ranges.

While in the coastal area the topographic levels are less than 100 m above sea level (m.a.s.l.), inland, and specifically in the northern sector of the Caroch Mountains, levels greater than 900 m.a.s.l. are reached. This marked altimetric difference is also appreciable in the climatology, thus, on the coast the average precipitation is around 480 mm, winters are mild with average temperatures above 10 ºC, and summers are hot and dry with average maximum temperatures of around 25 ºC. In the interior, on the contrary, annual precipitations are higher, reaching 500 mm and the thermal contrasts between summer and winter are more pronounced.

**Figure 1:**
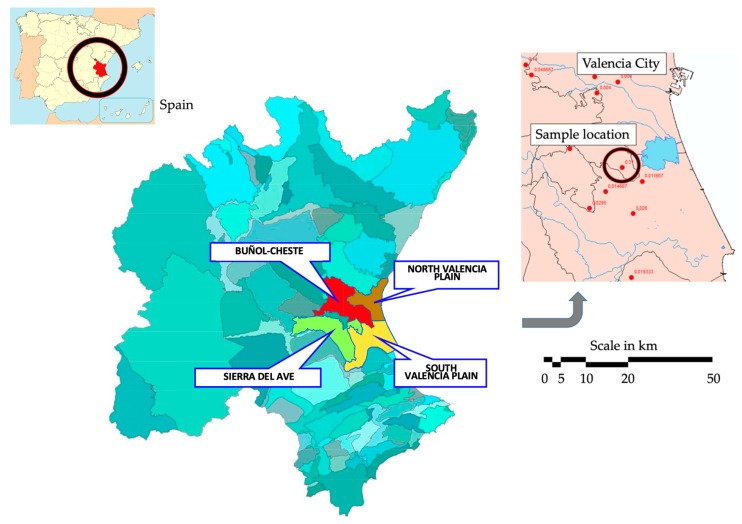
Location of the South Valencia Plain aquifer and the sample location.

The presence of pesticides has been detected in the waters of the Valencia Plain aquifer system [14]. This work is focused on improving knowledge about the use of pesticides in agriculture and its impact on groundwater quality. Figure 1 shows the location of the sample location inside the South Valencia Plain aquifer.

In the coastal area, where most of the population of the Valencian Region is concentrated, the city of Valencia itself and its metropolitan environment are located, with a highly developed economic activity in which industry and agriculture stand out. North and South Valencia Plain aquifers occupy an approximate area of 879.56 km^2^ coinciding with the coastal plain between the Mediterranean Sea and the Mesozoic reliefs that surround it (Sierras de Gátova and Náquera to the north, Sierra de La Rodana, Perenchiza, Besori and Caroch massif to the west and Sierra de Las Agujas to the south). The general flow pattern shows the existence of an underground flow in the west–east direction, towards the sea, coming from the most western areas, where the edge units that transfer their resources to the Plain are located, although they have frequent local exceptions to the regional context.

The piezometric levels vary between 60–70 m.a.s.l. at the north-western limit (La Eliana area and north of the Sierra Perenchiza) and sea level on the coast (areas of El Puig or the mouth of the new channel of the Turia river), with intermediate levels in central areas, as in the Picassent area where it is around 15 m.a.s.l. 

Annual piezometric fluctuations range from 10 m at the recharge edges and areas of greatest exploitation to 1 m at the eastern edge, coinciding with the discharge area. The hydraulic gradient (i) is extremely small, especially in the areas closest to the sea (0.01% < i < 0.0001%), although it may be locally modified by the condition generated by the concentration of farms, and also by the drainage caused by the Júcar river.

## 3. Materials and Methods

Some numerical models are available in scientific literature to approach organic chemicals’ fate and transport in the non-saturated zone. Table 2 shows a comprehensive list of numerical models for pesticide transport analysis with their main features.

In this work, simulations of terbuthylazine concentrations have been performed using two different numerical models, PESTAN and PRZM_GW. These two models have been chosen as they were specifically designed to model pesticide transport in the vadose zone and accounting to the fact that they do not need an extensive list of input data, this being one of the difficulties found when applying general 3D mass transport models to pesticide analysis. We should note too that PRZM-GW is the pesticide calculation groundwater module used by the more complex PWC model.

The Pesticide Analytical model (PESTAN) [15] was originally developed to understand the fate and transport of organic solute substances from the surface to finally reach the groundwater table. PESTAN provides a tool to perform evaluations when the environmental conditions are fairly unknown and not much preliminary data are available. In this common situation, the use of complex models is not justified as it may provide unreliable results. The formulation implemented in the model considers three mass transport mechanisms: (i) advection, (ii) dispersion and (iii) retardation by chemical reactions. Table 3 shows the main parameters and variables of the PESTAN model.

PESTAN results are obtained from the analytical solution of the 1D mass transport Equation (Equation (1)):

(1) $$\frac{\partial C}{\partial t}=D\frac{\partial^{2}C}{\partial x^{2}}-v\frac{\partial C}{\partial x}-\frac{\rho_{b}}{\theta }\frac{\partial S}{\partial t}-k_{l}C$$

The rate of loss of solute from liquid phase to solid phase due to sorption (∂S/∂t) and the change over time of the concentration of solute dissolved in water (∂C/∂t) are related through the Freundlich linear sorption coefficient K_d_ [20].

The analytical solution of Equation (1) is given by Equation (2):

(2) $$C\left(x,t\right)=\frac{C_{0}}{2}\exp\left(-k_{l}t\right)\left\{\mathrm{erf}\left[\frac{x+x_{0}-\frac{\mathrm{vt}}{R}}{2\sqrt{D_{t}/R}}\right]-\mathrm{erf}\left[\frac{x-\frac{\mathrm{vt}}{R}}{2\sqrt{D_{t}/R}}\right]\right\}$$

where R is the retardation factor defined as shown in Equation (3):

(3) $$R=1+\frac{\rho_{b}}{\emptyset }K_{d}$$

The Pesticide Root Zone Model for GroundWater (PRZM-GW) [16] was originally designed to simulate fate and transport of organic pollutants in the vadose zone within and immediately below the root zone. Its main difference to the simpler model PESTAN is that PRZM-GW considers specific formulations to analyze pesticide behavior in the vadose zone.

Vertical evolution of water content is computed by solving the 1D non-saturated flow equation given by Equation (4) (Richards equation):

(4) $$\frac{\partial \theta }{\partial t}=\frac{\partial }{\partial z}\left[K\left(\theta \right)\frac{\partial h}{\partial t}\right]$$

The total mass of pesticide distributed in time in each one of the three phases (solid, liquid and gas) are computed by solving the mass balance equations for the adsorbed, dissolved and gas phases shown in Equations (5) to (7):

(5) $$A\Delta z\frac{\partial \left(C_{w}\theta \right)}{\partial t}=J_{D}-J_{V}-J_{\mathrm{DW}}-J_{U}+J_{\mathrm{QR}}+J_{\mathrm{APP}}+J_{\mathrm{FOF}}\pm J_{\mathrm{TRN}}$$

(6) $$A\Delta z\frac{\partial \left(C_{S}\rho_{S}\right)}{\partial t}=-J_{\mathrm{DS}}-J_{\mathrm{ER}}$$

(7) $$A\Delta z\frac{\partial \left(C_{g}a\right)}{\partial t}=-J_{\mathrm{GD}}-J_{\mathrm{DG}}$$

Thanks to the formulation based on the mass distribution between phases, the model is able to provide an accurate characterization of the pesticide mass distribution over space and time.

Table 3 shows a comprehensive list of the main parameters and variables needed to run simulations in both models.

## 4. Case Study: Soil Contamination by Terbuthylazine in the Valencia Plain Aquifer (Spain)

Results for a real case-study when comparing outputs obtained by PESTAN and PRZM-GW with real terbuthylazine concentration data are shown below. Data were obtained from the official soil and groundwater control net of the Valencia Water Authority [14]. Soil and water samples were taken from agricultural land located at Picassent, 20 km southwest from Valencia City. This location was chosen as it showed a higher terbuthylazine concentration value than the other observation points of the net.

Currently in the study area, the use of the herbicide terbuthylazine is still allowed, while the compounds terbutrine and terbumetone were banned in 2003, their last authorized use being in 2007. The use of terbuthylazine has been decreasing in recent years, being replaced by the use of other herbicides, such as glyphosate. Terbuthylazine concentrations in groundwater, in recent years, have been below the reference limit of 0.1 µg/L, however, the products derived from desetil-terbuthylazine and terbumetone-desetil have reached values higher than 0.1 µg/L. 

A terbuthylazine concentration value in groundwater equal to 0.31 ppb was measured at the sample location. No information was available about the geological structure of soil and the characteristics of the pesticide application. Therefore, a whole set of scenarios was designed, accounting for different soil types which are common in the Valencia Plain aquifer and consider a range of terbuthylazine application doses (varying from 1 to 3 kg/ha). The simulations were done under a conservative perspective, so effects of volatilization and any degradation of the pesticide over the surface before its application to the soil were not included in the analyses.

Actual values of the rest of the parameters were considered in the simulations, including the annual average of the effective infiltration ratio (precipitation minus evapotranspiration), and the position of the phreatic surface.

**Figure 2:**
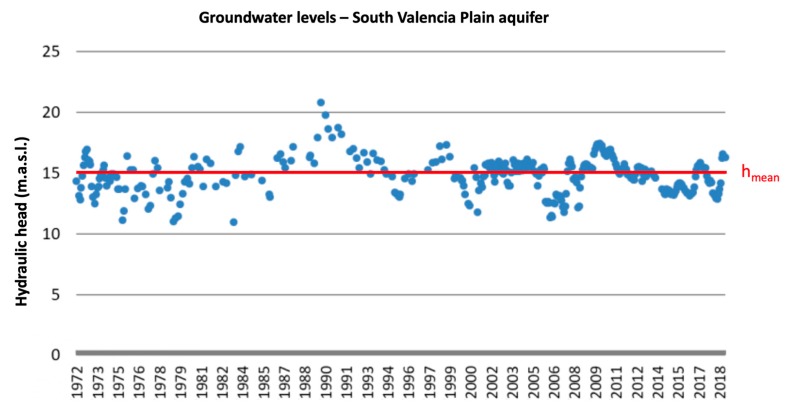
Evolution of piezometric levels (1972–2018) at the sample location.

Figure 2 shows the evolution over the period 1972–2018 of the piezometric levels in the sample location. Piezometric levels at the sample location can be considered to be stable over time (h_mean_ = 15 m.a.s.l.), that is 5 m below the topographic surface. 

Therefore, in order to properly consider the characteristics of the soil and its geological structure in the simulation process, five different types of soil were considered, therefore defining different simulation scenarios based on soil type, application type and dose value. For each one of these soil types, the characteristic parameters were also defined, following recommendations given by the model manuals. Therefore, specific values of soil density, saturation, characteristic curve coefficient, saturated hydraulic conductivity, organic carbon content and sand and clay contents have been fixed for every simulation scenario. Table 4 shows the parameter values included in the modelling process, the soil characteristics and the value of the different parameters included in PESTAN and PRZM-GW.

Therefore, for each one of these five soil types, a single dose application and different annual applications of terbuthylazine were considered, both at 3 m and 5 m depth. The values of terbuthylazine-simulated doses were 1 kg/ha, 2 kg/ha and 3 kg/ha. Therefore, a total number of 60 simulations scenarios were modeled using PESTAN and PRZM-GW.

## 5. Results

### 5.1. Terbuthylazine Concentrations Computed by PESTAN

**Figure 3:**
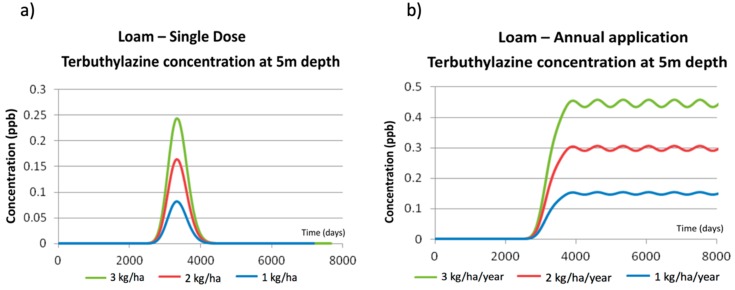
PESTAN terbuthylazine concentrations in loamy soil at 5 m depth. (**a**) Single dose application. (**b**) Annual application.

**Figure 4:**
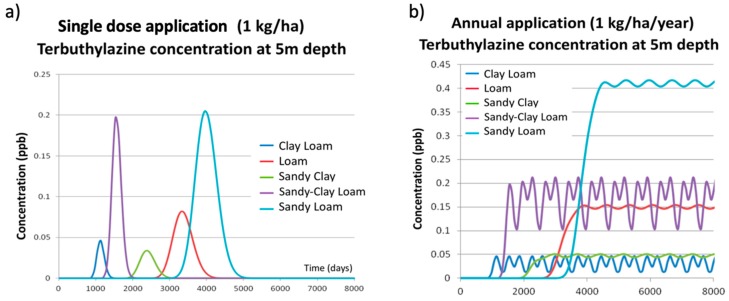
PESTAN terbuthylazine concentrations at 5 m depth for different types of soil. (**a**) Single dose application. (**b**) Annual application.

PESTAN results analysis has been done by comparing the values of terbuthylazine concentrations in groundwater obtained for the different scenarios (considering every soil type and every application pattern). Figure 3 and Figure 4 show the terbuthylazine breakthrough curves obtained by PESTAN under the different simulated scenarios.

Figure 3a shows the effect of increasing the terbuthylazine dose from 1 kg/ha to 3 kg/ha on the final concentrations observed on groundwater at 5 m depth. The prediction of the model is such that the date on which the maximum value is observed is the same for the three scenarios. Besides, the model provides results that are just proportional to the single dose value, so the peak value of the concentration curve is proportional to this dose.

Figure 3b shows the values of terbuthylazine concentrations in groundwater at 5 m depth simulated by PESTAN for annual applications at the same rates (1 kg/ha to 3 kg/ha). When comparing these results with the ones shown in Figure 3a, two effects are observed:

- When applying an annual dose, the impact of terbuthylazine over groundwater quality is initially observed at the same time as the one detected for a single dose (day = 2900 in Figure 3a) but the maximum values of the concentrations curve are totally different. When terbuthylazine is applied annually, the maximum value of the breakthrough curve is observed periodically and its value doubles the ones obtained when applying a single dose. For example, for a single dose equal to 2 kg/ha, the maximum terbuthylazine concentration value is 0.16 ppb, while this value is equal to 0.31 ppb when there is an annual application.
- The shapes of the breakthrough curves obtained for both types of applications are totally different. While for a single dose (Figure 3a) the breakthrough curve initially increases until reaching a maximum value to finally decrease down to zero, for an annual application (Figure 3b) it has been observed that, once the maximum value has been reached, it slightly oscillates but the concentrations are never significantly decreasing so long as the application is maintained in time.

These results, obtained for loamy soil, cannot be generalized and used for every other soil type considered in the simulation process. To visualize and understand the effect of soil type, the corresponding simulations have been done using PESTAN, keeping the same dose value and application patterns. Results are shown in Figure 4.

Figure 4a shows the breakthrough curves of terbuthylazine concentrations in groundwater at 5 m depth obtained by PESTAN when a single dose application of 1 kg/ha is used. Results clearly show the following effects:

- For the same terbuthylazine dose, the breakthrough curve shapes are similar in every simulation scenario as they all show a maximum value and symmetric distribution.
- The maximum concentration value is highly dependent on the simulation scenario.
- The initial time for which the effect of terbuthylazine is first detected in the groundwater is highly variable, covering a range from 900 days (clay-loam) to 3000 days (sandy-loam).

Figure 4b shows the breakthrough curves of terbuthylazine concentrations in groundwater at 5 m depth obtained by PESTAN when an annual application of 1 kg/ha is used. Some similarities and differences are found from the ones corresponding to a single dose:

- The breakthrough curve shapes are all similar for every soil type as they all reach a maximum value and oscillate around it while the application continues.
- The maximum value is highly dependent on the simulation scenario.
- The magnitude of the oscillation is also highly dependent on the simulation scenario. A maximum oscillation value of 0.11 ppb has been found for the sandy-clay-loam soil type.
- The initial time for which the effect of terbuthylazine is first detected in the groundwater is also highly variable, covering a range from 900 days (clay-loam) to 3000 days (sandy-loam). For every annual application scenario, this initial time is the same that was observed on the single dose application scenarios.

**Figure 5:**
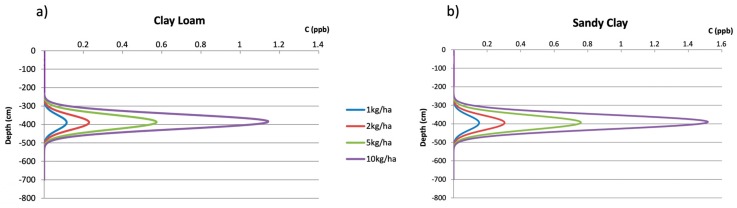
PESTAN terbuthylazine concentrations at t=2000 days for two types of soil and different single dose concentrations. (**a**) Clay-loam. (**b**) Sandy-clay.

PESTAN analytical formulation also allows obtaining results in terms of pesticide concentration in the dissolved phase with respect to vertical depth. In order to understand and visualize the impact of soil characteristics, Figure 5 shows terbuthylazine concentrations at t = 2000 days after a single dose (1 kg/ha to 10 kg/ha) has been applied to two different soil types (clay-loam and sandy-clay). Maximum single terbuthylazine doses have been increased from 3 kg/ha to 10 kg/ha in order to better visualize those results obtained by the PESTAN model.

As shown in Figure 5 the evolution in time of terbuthylazine with respect to vertical depth shows two main characteristics:

- The position of the maximum value of pesticide concentration after 2000 days (z = 390 cm) is the same for every soil type. It has been seen that the only parameter which affects the position of the maximum value of the pesticide concentration is the effective infiltration rate.
- Concentration values distribute symmetrically from the centered-maximum pesticide value. Soil characteristics affect this maximum concentration value (1.15 ppb for clay-loam and 1.55 ppb for sandy-clay when a 10 kg/ha single terbuthylazine dose is applied). Concentration values are therefore dependent from every soil characteristic shown in Table 4.

Similar results as those shown in Figure 5 for clay-loam and sandy-clay have been obtained for every other soil type considered in the PESTAN simulations.

### 5.2. Terbuthylazine Concentrations Computed by PRZM-GW

**Figure 6:**
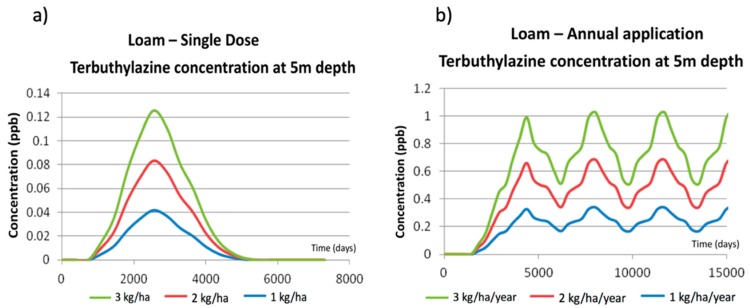
PRZM-GW terbuthylazine concentrations in loamy soil at 5 m depth. (**a**) Single dose application. (**b**) Annual application.

**Figure 7:**
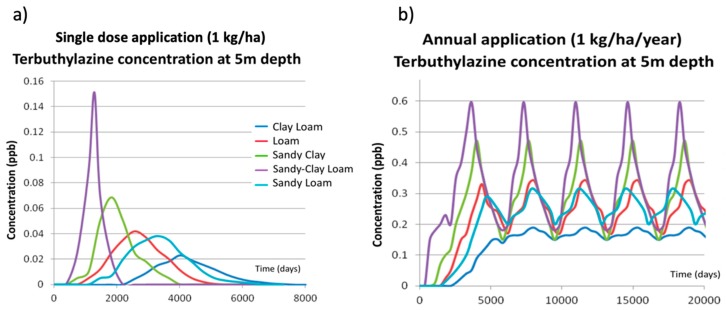
PRZM-GW terbuthylazine concentrations at 5 m depth for different types of soil. (**a**) Single dose application. (**b**) Annual application.

Using the same approach as the one explained in Section 5.1, PRZM-GW results analysis has also been done, comparing the values of terbuthylazine concentrations in groundwater obtained for every scenario. Figure 6 and Figure 7 show the terbuthylazine breakthrough curves obtained by PRZM-GW under the different simulated scenarios. 

Figure 6a shows the effect of increasing the terbuthylazine dose from 1 kg/ha to 3 kg/ha on the final concentrations observed on groundwater at 5 m depth. The prediction of the model is such that the date in which the maximum value is observed is the same for the three scenarios. Besides, the model provides results that are just proportional to the single dose value, so the peak value of the concentration curve is proportional to this dose.

These patterns are similar to those obtained when using PESTAN. However, the maximum concentration values are reduced to 50% of the maximum values obtained by PESTAN under the same conditions. This effect may be due to the fact that PRZM-GW distributes total terbuthylazine mass in the three phases (solid, liquid and gas) while PESTAN only considers the distribution in the liquid phase. Furthermore, not only the maximum values of pesticide concentration in water are different, but also the shape and position of the breakthrough curves.

A comparison between Figure 3a and Figure 6a shows that the breakthrough curve obtained by PESTAN is delayed with respect to the one obtained by PRZM-GW, so the times corresponding to the maximum concentration value of terbuthylazine in groundwater are obtained sooner by PRZM-GW (2300 days) than by PESTAN (3400 days). Breakthrough curves obtained by PRZM-GW are also symmetric with respect to the maximum value, as it was obtained by PESTAN, but the time period in which the effect of terbuthylazine is observed to be affecting groundwater quality (range observed in the time axis) is much higher in the results obtained by PRZM-GW (4000 days) than by PESTAN (1100 days).

Figure 6b shows the values of terbuthylazine concentrations in groundwater at 5 m depth simulated by PRZM-GW for annual applications at the same rates (1 kg/ha to 3 kg/ha). When comparing these results with the ones shown in Figure 6a, two effects are observed:

- When applying an annual dose, the impact of terbuthylazine over groundwater quality is initially observed at the same time as the one detected for a single dose (day = 1000 in Figure 5a) but the maximum values of the concentrations curve are totally different. When terbuthylazine is applied annually, the maximum value of the breakthrough curve is observed periodically and its value is almost one order of magnitude higher than the ones obtained when applying a single dose. For example, for a single dose equal to 2 kg/ha the maximum terbuthylazine concentration value is 0.082 ppb, while this value is equal to 0.65 ppb when there is an annual application. This effect was also observed on results obtained by PESTAN, but the differences found between results for different application patterns (single dose or annual application) were not so dramatic.
- The shapes of the breakthrough curves obtained for both types of application are totally different. While for a single dose (Figure 6a) the breakthrough curve initially increases until reaching a maximum value to finally decrease down to zero, for an annual application (Figure 6b) it has been observed that it takes a much longer time to reach the maximum value, and, once this maximum value has been reached, it oscillates largely in time and value. These oscillations are now much more significant than the ones observed in results provided by PESTAN (Figure 3b). It has been observed that the difference between the maximum and minimum terbuthylazine concentrations in groundwater once the oscillation phase starts (range in the vertical axis of Figure 6b) decreases pesticide concentration by a factor of 2 (for an annual application of 2 kg/ha/year maximum concentration value is equal to 0.7 ppb while minimum value in the oscillation phase is 0.35 ppb).

A comparison between Figure 3b and Figure 6b show that the breakthrough curve obtained by PRZM-GW detects pesticide in groundwater sooner than the one obtained by PESTAN. However, both breakthrough curves show the maximum terbuthylazine concentration at a similar time (4000 days). The maximum concentration value is much higher on results obtained by PRZM-GW than the ones obtained by PESTAN for an annual application. For example, if an annual application of 2 kg/ha/year is considered, the maximum concentration of terbuthylazine in groundwater obtained by PRZM-GW (0.70 ppb) is more than 100% higher than the results obtained by PESTAN (0.31 ppb). This effect has been observed for every other dose considered in the simulations.

As it was said before, these results, obtained from loamy soil, cannot be generalized and used for every other scenario considered in the simulation process. To visualize and understand the effect of soil type, the corresponding simulations have been done using PRZM-GW. Results are shown in Figure 7.

Figure 7a shows the breakthrough curves of terbuthylazine concentration in groundwater at 5 m depth obtained by PRZM-GW when a single dose application of 1 kg/ha is used. Results clearly show the following effects:

- For the same terbuthylazine dose, the breakthrough curve shapes are similar for every soil type as they all show a maximum value and a symmetric distribution.
- The maximum concentration value is highly dependent on the soil type.
- The initial time for which the effect of terbuthylazine is first detected in the groundwater is highly variable, covering a range from 900 days (clay-loam) to 3000 days (sandy-loam).

These effects obtained by PRZM-GW are similar to those observed when using PESTAN. However, the differences between the results obtained by these two models can be seen by comparing Figure 4a and Figure 7a. PESTAN results show that, depending on the soil type, the effect of terbuthylazine in groundwater quality extends from 900 days to 5000 days (Figure 4a) while PRZM-GW results show that this time range varies from 500 days to 8000 days (Figure 7a). Therefore, effects of terbuthylazine in groundwater predicted by PRZM-GW are detected sooner and last longer in time than those predicted by PESTAN.

Figure 7b shows the breakthrough curves of terbuthylazine concentration in groundwater at 5 m depth obtained by PRZM-GW when an annual application of 1 kg/ha is used. As it was observed when analyzing PESTAN results, some similarities and differences are found from the ones corresponding to a single dose:

- The breakthrough curve shapes are all similar for every soil type as they all reach a maximum value and oscillate around it while the application continues. However, the magnitude of this oscillation is much higher than the corresponding ones obtained by PESTAN.
- The maximum value is highly dependent on the soil type. Maximum values of terbuthylazine concentrations in groundwater have been obtained for sandy-clay-loam soil type (reaching a peak value equal to 0.6 ppb for an annual application of 1 kg/ha/year).
- The magnitude of the oscillation is also highly dependent on the soil type. A maximum oscillation value of 0.42 ppb has been found for the sandy-clay-loam soil type. This oscillation is almost four times higher than the one predicted by PESTAN for the same soil type.
- The initial time for which the effect of terbuthylazine is first detected in the groundwater is highly variable, covering a range from 500 days (sandy-clay-loam) to 2000 days (clay-loam). For every annual dose application scenario, this initial time is the same one that was observed in the single dose application scenarios.

The difference between the results obtained by these two models when annual doses are applied can be seen by comparing Figure 4b and Figure 7b. PESTAN results show that, depending on the soil type, the concentration of terbuthylazine in groundwater varies from 0.01 ppb (clay-loam soil) to 0.42 ppb (sandy-loam soil). However, these ranges are much higher than the predictions made by PRZM-GW. PRZM-GW results show that, depending on the soil type, the concentration of terbuthylazine in groundwater varies from 0.19 ppb (clay-loam soil) to 0.60 ppb (sandy-clay-loam soil). Therefore, both models obtain different maximum and minimum values of terbuthylazine concentrations for different types of soil and they differ greatly on their corresponding predictions.

## 6. Discussion

**Figure 8:**
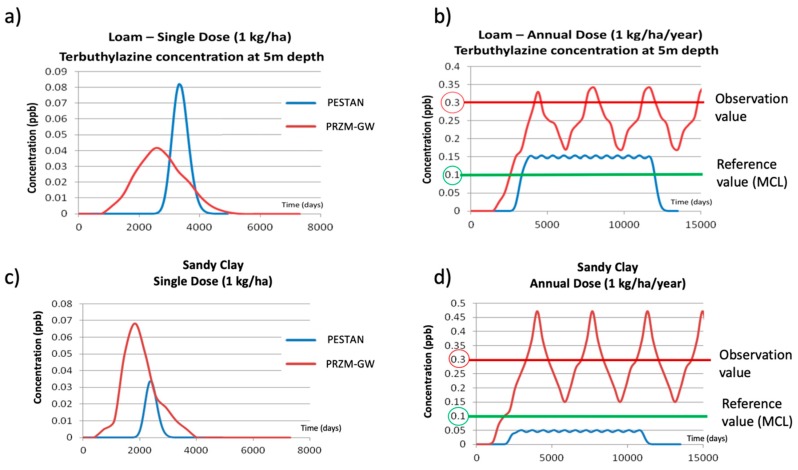
Simulation results for different soils. (**a**) Loamy soil. Single dose. (**b**) Loamy soil. Annual application (**c**) Sandy-clay soil. Single dose. (**d**) Sandy-clay soil. Annual application.

To illustrate the differences between results obtained by both models and to compare the actual observations with the predictions made by PESTAN and PRZM-GW, Figure 8 shows the comparison between the results obtained by PESTAN and PRZM-GW for four different simulation scenarios: two types of soils (loamy soil and sandy-clay soil) and two terbuthylazine application patterns (single dose of 1 kg/ha and annual application of 1 kg/ha/year).

Results for scenarios considering loamy soil and sandy-clay soil when a single terbuthylazine dose of 1 kg/ha is applied are shown in Figure 8a,c. The maximum terbuthylazine concentration values obtained by the models for a loamy soil are 0.08 ppb (PESTAN) and 0.04 ppb (PRZM-GW). However, for sandy-clay soil, these maximum values are 0.07 ppb (PRZM-GW) and 0.035 ppb (PESTAN). The shape and position of the breakthrough curves are also very much dependent on the simulation scenario. Results show that PRZM-GW always predicts the appearance of the maximum value of the breakthrough curve before the prediction made by PESTAN, no matter what the actual value of the maximum value is.

**Figure 9:**
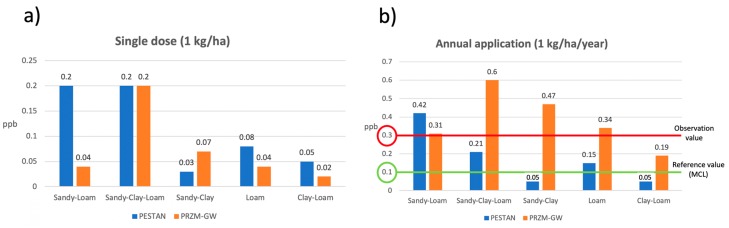
Maximum terbuthylazine concentration values. (**a**) Single dose (1 kg/ha). (**b**) Annual application (1 kg/ha/year).

Results for loamy soil and sandy-clay soil scenarios when an annual application of terbuthylazine of 1 kg/ha/year is applied are shown in Figure 8b,d. The observed terbuthylazine concentration value in groundwater at 5 m depth (0.3 ppb) and the Maximum Concentration Level (MCL), which is the legal reference value in Valencia Province (0.1 ppb) are also shown. Figure 9 shows the results obtained by PESTAN and PRZM-GW in terms of the maximum terbuthylazine concentrations in groundwater obtained for every scenario, considering 1 kg/ha as the pesticide dose.

Figure 9a shows that terbuthylazine concentrations obtained with PRZM-GW are higher only for a sandy-clay soil type if the pesticide is applied on a single dose. Therefore, for a single dose application, no conclusions can be made a priori about the behavior of terbuthylazine, even if the soil type and the dose value are known and specific modeling should be done on every single case.

However, Figure 9b shows that if the application (1 kg/ha/year) is done annually, concentration values obtained by PRZM-GW are higher than those obtained by PESTAN for every soil type except for sandy-loam. Results show that predictions made by PRZM-GW lead to very high concentrations of terbuthylazine if an annual application pattern is considered in comparison with concentrations obtained for a single dose. 

**Figure 10:**
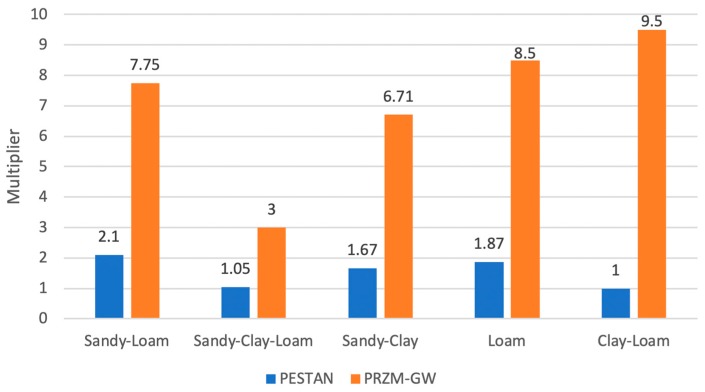
Multiplier values for a 1 kg/ha (annual application vs single dose).

Figure 10 shows the comparison between the maximum concentration values obtained for the single dose and annual application scenarios for every soil type. The comparison is shown in terms of the multiplier value, that is, the number of times that the annual application maximum concentration is higher than the same value for the single dose scenario.

The maximum multiplier value has been obtained for the clay-loam soil type, where the concentrations obtained for annual applications were 9.5 times higher than dose obtained for the single dose. These multiplier values obtained by PRZM-GW are much higher than the ones obtained when using PESTAN, for which the maximum multiplier value is 2.1 (sandy-loam). As was said before, no conclusions can be made a priori about the behavior of terbuthylazine, even if the soil type and the application patterns are known and specific modeling should be done on every single case.

Despite PRZM-GW being a more complex model than PESTAN, results obtained by PESTAN such as the ones shown in Figure 5 cannot be provided by PRZM-GW, which focuses on simulating pesticide concentrations only on the saturated zone of the aquifer.

The comparison of those results obtained by PESTAN and PRZM-GW with the current situation and terbuthylazine observations in the study area lead to the following final remarks:

- Terbuthylazine concentrations in groundwater observed in the Picassent agricultural land (0.3 ppb) are three times higher than the Maximum Concentration Value (MCL = 0.1 ppb) considered by the regulatory agency.
- This concentration value cannot be reached by any one of the single dose values considered in the simulation process, no matter which one of the models (PESTAN or PRZM-GW) are used.
- Collected data show that the groundwater table is located almost invariantly at 5 m depth. Therefore, no sensitivity analysis about this parameter has been performed.
- As no information about the soil characteristics was available, a sensitivity analysis about soil type has been done. Results show that PRZM-GW is able to reproduce the observed terbuthylazine concentrations considering reasonable values of pesticide annual applications for different soil types.
- Results obtained by PRZM-GW demonstrate that annual applications of terbuthylazine around 1 kg/ha/year are being applied in the Picassent agricultural area, leading to pesticide concentrations 300% higher than the MCL. To obtain the same final concentration values, PESTAN applications should be even higher than those given by PRZM-GW, even leading, in some cases, to some unrealistic pesticide application values.

Therefore, in order to improve groundwater quality and reduce terbuthylazine concentrations to a value lower than the legal MCL, annual terbuthylazine applications should be reduced to at least 1/3 of the current ones. Once the models are available and ready to simulate, it is also important to perform a detailed soil analysis campaign in order to properly identify soil parameters and therefore reduce the uncertainty of results. Further analysis using more complex models which consider vertical parameter heterogeneity should only be done when all the information about soil properties is available.

## 7. Conclusions

This paper shows, for the first time in literature, numerical modeling results from an analysis of groundwater pollution by terbuthylazine on the Valencia South Plain aquifer. This work has been developed using pesticide-specific models and answers to the necessity of performing pesticide transport simulations, leading to a better understanding of the behavior of these contaminants considering the scarce available information. Results can be used to establish limits to pesticide applications in agricultural activities in the Valencia Plain aquifer.

In this work, two different numerical models (PESTAN and PRZM-GW) have been applied to assess groundwater quality simulating the transport of terbuthylazine in the soil. A case-study in Valencia Province (Spain) where actual values of terbuthylazine concentration in groundwater were available has been presented. Despite both models being able to simulate terbuthylazine concentrations in groundwater, only PRZM-GW provides accurate results in comparison with actual observations in the Valencia Province case. 

An extensive analysis of the possibilities of using both models have been presented, taking into account the influence of five different soil types and six different pesticide application patterns. 

For single dose applications, PESTAN simulation results show that the breakthrough curve shapes are all similar for every scenario, showing a maximum value and a symmetric distribution. The maximum value and the initial time for which the effect of terbuthylazine is first detected in the groundwater is also highly dependent on the soil type and application pattern. The same conclusions have been obtained when using PRZM-GW. However, effects of terbuthylazine in groundwater predicted by PRZM-GW are detected sooner and last a longer time than those predicted by PESTAN. 

For annual applications, PESTAN simulation results show that the breakthrough curve shapes are all similar for every scenario and they all reach a maximum value and oscillate around it while the application continues. The maximum value and the magnitude of the oscillation is highly dependent on the soil type and application pattern. However, the breakthrough curve obtained by PRZM-GW detects pesticide in groundwater sooner than those obtained by PESTAN. Both breakthrough curves show the maximum terbuthylazine concentration at a similar time but the maximum concentration value is much higher in results obtained by PRZM-GW than the ones obtained by PESTAN.

Despite PRZM-GW being a more complex model, PESTAN is able to simulate pesticide concentration values at any vertical depth inside the vadose zone as a function of time. These types of results cannot be provided by PRZM-GW, which focuses on simulating pesticide concentrations only on the saturated zone of the aquifer. 

It has been seen that for long-term simulations, PESTAN predictions underestimate the concentration of terbuthylazine and they may even provide predictions which are under the MCL. On the other hand, predictions made by PRZM-GW are much more accurate than those obtained by PESTAN. PRZM-GW obtains good results when reproducing the observed values of terbuthylazine concentration in two types of soil (loamy soil and sandy-clay).

## Figures and Tables

**Table 1:** Terbuthylazine physicochemical properties.

Property	Value
Vapor pressure	0.15 mPa at 25 °C
Volatility	0.014 mg/m^3^ at 20 °C
Density	1.188 at 20 °C
Octanol–water partition coefficient (K_ow_)	1096
Solubility in water	8.5 mg/L at 20 °C

**Table 2:** Main features of available numerical models for pesticide analysis.

Model	References	Objetives	NSZ ^(^*^)^	SZ ^(^**^)^	1D-2D-3D	Processes
PESTAN	[15]	Pesticide concentration in soil	✓		1D	Advection, dispersion and reactions
PRZM_GW	[16]	Pesticide concentration in soil and groundwater	✓	✓	1D	Advection, dispersion, reactions and root interactions
PWC	[17]	Pesticide concentration in soil, surface water and groundwater	✓	✓	1D	Advection, dispersion, reactions and root interactions
TOXSWA	[18]	Pesticide concentration in aquatic ecosystems	✓		2D	Advection, dispersion, diffusion, adsorption, and volatilization, advection
PEARL	[19]	Leaching of pesticides to GW drainage to SW and soil persistence	✓	✓	1D	Advection, dispersion, adsorption, volatilization, transformation, ETP and root interaction

^(^*^)^ NSZ: Non-Saturated Zone; ^(^**^)^ SZ: Saturated Zone.

**Table 3:** PESTAN and PRZM-GW main parameters and variables.

Model	Parameter/Variable	Description	Units
**Both**	t	Time	T
	θ	Volumetric water content (volume of pore water / total volume of sample)	-
	Cw or C	Concentration of contaminant dissolved in water	M/L^3^
**PESTAN**	x	Distance along the flow path	L
	D	Longitudinal dispersion coefficient	L^2^/T
	v	Linear average flow velocity (pore water velocity)	L/T
	$\rho_{b}$	Soil solid phase bulk density	M/L^3^
	S	Concentration of pollutant in the solid phase (mass of pollutant in soil/mass of soil)	M/M
	k_1_	First-order decay coefficient in the liquid phase	T^−1^
	∅	Soil total porosity	-
**PRZM-GW**	K(θ)	Hydraulic conductivity under non-saturated conditions	L/T
	h	Total hydraulic potential	L
	A	Transversal section of the soil column	L^2^
	Δz	Depth	L
	C_S_	Concentration of contaminant in soil	M/M
	C_G_	Concentration of contaminant in gas phase	M/L^3^
	a	Volumetric air content in soil	L^3^/ L^3^
	$\rho_{S}$	Soil density	M/L^3^
	J_D_	Mass flux due to dispersion and diffusion in the dissolved phase	M/T
	J_V_	Mass flux due to advection in the dissolved phase	M/T
	J_GD_	Mass flux due to dispersion and diffusion in the gas phase	M/T
	J_DW_	Mass flux due to degradation in dissolved phase	M/T
	J_DG_	Mass flux due to degradation in the gas phase	M/T
	J_U_	Mass flux from the dissolved phase due to root uptake	M/T
	J_QR_	Mass flux from runoff	M/T
	J_APP_	Mass flux from pesticide application to soil	M/T
	J_FOF_	Mass flux given from the crops to the soil	M/T
	J_DS_	Mass flux due to the chemical degradation of adsorbed contaminant	M/T
	J_ER_	Mass flux (loss) by dissolution or sediments erosion	M/T
	J_TRN_	Mass flux due to other reactions	M/T

**Table 4:** Parameter values and characteristics of the different types of soil considered in the simulations.

	Parameter	Sandy-Loam	Sandy-Clay-Loam	Sandy-Clay	Loam	Clay-Loam
**Soil-Specific Parameters**	$\rho_{b}$ (g/cm^3^)	1.335	1.47	1.28	1.47	1.28
	Saturation	0.435	0.42	0.426	0.451	0.476
	Characteristic curve Coeff.	4.9	7.12	10.4	5.39	8.52
	K_sat_ (cm/h)	4.42	1.31	0.12	1.04	0.26
	f_oc_ (%)	0.71	0.19	0.38	0.52	0.1
	Sand (%)	65	60	50	40	35
	Clay (%)	35	40	50	60	75
**Common Parameters**	Groundwater temperature	17 °C				
	Terbuthylazine water solubility	6.5 mg/L				
	Half life	60 days				
	K_oc_	220 mg/L				
	Effective infiltration rate	1030 mm/year				
	Phreatic level depth	5.0 m				
	Root depth	20 cm				
	Maturity date	Day 5 of Month 12				
	Harvest date	Day 10 of Month 12				
	Screen length	1 m				
	Albedo	0.2				
